# Who shouts the loudest? A qualitative study exploring barriers and enablers to implementing a low emission zone in a Northern UK city

**DOI:** 10.1016/j.trip.2024.101112

**Published:** 2024-05

**Authors:** Cathy Knamiller, Rukhsana Rashid, Maria Bryant, Emma Bailey, Rosemary R.C. McEachan

**Affiliations:** a Bradford Institute for Health Research, Bradford Teaching Hospitals NHS Foundation Trust, Bradford, West Yorkshire BD9 6RJ, United Kingdom; b Department of Health Sciences, University of York, Heslington, York, West Yorkshire, YO10 5DD, United Kingdom; c Hull York Medical School, University of York, Heslington, York, West Yorkshire, YO10 5DD, United Kingdom

**Keywords:** Low emission zone (LEZ), Clean air zone (CAZ), UK, Barriers, Enablers, Policy implementation, Health

## Abstract

Pollution is a major cause of ill health globally. Low emission zones (LEZ) have been identified as effective in reducing pollution and are increasing in popularity but remain divisive. Understanding what factors help or hinder implementation is important. In the UK, Clean Air Zones (CAZ, a type of LEZ) are being implemented in several cities. We aimed to identify key barriers and enablers to the implementation of a CAZ in real time, as policy was being developed and implemented in a large Northern city in England, UK. Twenty-five semi-structured interviews were conducted with city stakeholders and implementors approximately 6 months before a CAZ charging non-compliant taxis, buses, heavy goods vehicles and vans was launched. Thematic analysis was used to analyse data. Implementers were required to operate within a tight policy framework. Key enablers included: freedom to adapt the framework to local context, financial support, and cross-sector working. A focus on health was felt to be useful in justifying the policy to the public. Key barriers included conflict and opposition from local industry, politicians, and communities. Implementation of air quality policy which involves traffic restrictions remains controversial. The voices which ‘shout the loudest’ are often those with negative views, and these can create divisive discourse which shape public opinion and damage confidence of implementers. A systems perspective is needed to understand socio-political contexts which can influence implementation success. We provide recommendations to other areas considering implementing a LEZ.

## Highlights

• Reports barriers and enablers to LEZ implementation in real time in a large UK city.
• Cross-sector working incorporating transport, planning and health was key enabler.
• Political conflict was a barrier; vocal anti-LEZ campaigns shaped public opinion.
• Using health messages to justify LEZ implementation improved public acceptability.
• We provide recommendations for others considering implementing this policy.

## Introduction

Air pollution is the greatest environmental threat to human health worldwide (World Health Organisation, 2021). It causes a range of acute (e.g. breathing difficulties) and chronic (stroke, respiratory and cardiovascular) conditions, reducing life expectancy (Public Health England, 2018). Pollution related ill-health costs across Europe are estimated to be €166 billion per year (de Bruyn and de Vries, 2020). Older people, children, those with pre-existing lung and heart conditions, and people on lower incomes are most at risk of the negative health impacts of pollution (Public Health England, 2018). Typically, areas with higher levels of deprivation tend to be areas with higher levels of pollution (Barnes et al., 2019), which can exacerbate the health inequalities of already vulnerable communities living in these areas (Fecht et al., 2015).

Low emission zones (LEZ) have been identified as a potentially effective intervention to reduce pollution levels in cities (Chamberlain et al., 2023). Typically, these work by restricting the access of older, more polluting vehicles, defined by their emissions standards at point of construction, within predefined geographic regions. In 2022 there were 320 active LEZ across Europe and this is expected to increase to 507 by 2025 (Azdad et al., 2022).

The practice of implementing a policy such as a LEZ is highly complex and despite their growing popularity, there is little published empirical research which explores factors which help or hinder implementation. In wider literature, McTigue et al. (McTigueet al., 2020, McTigueet al., 2018) identify a range of key factors related to successful implementation of transport policy, including clear policy documentation, availability of resources, inter-organisation support and communication, and policy champions (2020, 2018). Pfadenhauer et al (2017) further elaborate on the importance of *context* elaborating seven contextual domains (geographical, epidemiological, socio-cultural, socio-economic, ethical, legal, and political contexts), each of which impacts implementation at multiple levels. These frameworks provide a useful lens to understand what factors may influence implementation of policies such as LEZ.

In response to failing to achieve the annual mean limit value of Nitrogen Dioxide (NO_2_) as set out in the EU’s Ambient Air Quality Directive (2008/50/EC) in a number of areas, the UK Government directed a number of local authorities to implement a charging clean air zone (CAZ, a form of LEZ) in 2018 (McEachan et al., 2022). The Clean Air Zone framework (Department for Environment, Food & Rural Affairs, and Department for Transport, 2017) outlined four possible types of charging Clean Air Zones varying in levels of restriction, which would see charges for vehicles not compliant with Euro 4 standards (for petrol) or Euro 6 standards (for diesel).^1^

The realisation of the CAZ framework in England has proved to be contentious (Tyers and Smith, 2023). Substantial delays have been experienced in many areas, with some pulling out entirely (Leeds City Council, 2020, Greater Manchester Combined Authority, 2022). The current study aims to explore barriers and enablers to the policy in ‘real-time’ as it was being implemented, but before it was formally launched, and to provide lessons learned for other areas wishing to implement this approach.

## Methods

### Design

We conducted a qualitative study using semi-structured interviews with key stakeholders to explore the barriers and enablers to the development of a CAZ during its pre-implementation phase, defined as the period after the plans for the CAZ were approved, but before it was launched. The study was approved by Bradford Leeds NHS Research Ethics Committee on the 30th of June 2020 (20/YH/0158). We follow the consolidated criteria for reporting qualitative research (Tong et al., 2007). The study is part of a larger project which aims to evaluate the health and economic impact of the Bradford CAZ (McEachan et al., 2022).

### Setting

Bradford is a multicultural city in the North of England. It is the 7th largest metropolitan district in England with a population of more than 546,400 (Office for National Statistics, 2023). Fifty-seven percent of the population consider themselves White British, 32 % Asian, 4 % White other and 2 % Black (Office for National Statistics, 2023). Bradford is a socio-economically deprived city with 34 % of residents living in areas that rank in the most deprived quintile of local areas in England (Ministry of Housing Communities and Local Government, 2019). There are 4 air quality management areas in the city and 5 further areas of concern in the city. Key pollutants of concern are NO_2_ and particulate matter, with traffic a significant source, in addition to industry, heat and power generation, domestic sources and natural activities (City of Bradford Metropolitan District Council, 2023). Geographically, the areas with the highest levels of pollution in Bradford tend to be those which are more deprived (Mueller et al., 2018).

In 2018 Bradford was directed by the Government Joint Air Quality Unit to develop plans to implement a charging clean air zone, as a number of areas of the city exceeded the legal limit of 40 µg/m^3^ of Nitrogen Dioxide (NO_2_). (McEachan et al., 2022) After modelling various options, a charging ‘CAZ C’ class (charging for buses, coaches, taxis, private hire vehicles, heavy goods vehicles, vans, minibuses) with additional requirements for private hire vehicles and taxis to be hybrid or electric was approved in February 2020, and after some delays, was launched on 26th September 2022.

### Sample & procedure

Participants were purposively selected based on their involvement or influence on the development of the CAZ using a sampling frame, including members of the board tasked within development and implementation. This group included representation from health, highways, economic development, procurement, the grants team, marketing, and research. Participants were recruited via snowball sampling was used to identify further participants including pro and anti-CAZ local councillors and action groups, and a taxi lobby group. Introductions were made through email and followed up by the researcher.

We planned to conduct 20 interviews, estimating that this would give us a rich dataset, whilst balancing realistic research constraints (King, 2021). Participants were emailed an information sheet and asked to provide informed consent prior to interviews which were recorded via video-conferencing or zoom. Interviews took place between January − April 2022, just after an expected launch date of January 2022 had been delayed, and at a time where no official launch date had been set. Interviews were conducted by CK or RR following a semi-structured interview guide (see Supplemental file 1) and lasted no more than an hour. Key topics included stages of CAZ development, public consultation, and marketing, the specification of the CAZ, the impact of the COVID pandemic and expected outcomes. To sense check, the topic guide was piloted with one health researchers and one council employee (King, 2021). Interviews were recorded, anonymised, and transcribed verbatim.

The first author (CK) was employed as a research fellow at the time, working on projects related to air quality and health. CK has a PhD and several years’ experience conducting and analysing qualitative research. She has lived and worked in Bradford for 18 years. A small number of interviewees were previously known to her because of this.

### Analyses

Transcripts were analysed using thematic analysis. Themes were constructed using an inductive, semantic approach and a coding framework developed which was continually updated during the process of coding (Braun and Clarke, 2006). Nvivo (release 1.6.1, QSR International) was used to organise data. Initial coding was first independently carried out by CK and RR and a final coding framework was jointly developed by CK, EB and RM. CK and EB then double coded all interviews according to this framework. The frameworks of McTigue et al. (2020) and Pfadenhauer et al. (2017) were used as a lens to interpret the themes emerging from the data and ascertain a deeper understanding of the factors which helped and hindered implementation.

## Results

Of the 34 potential interviewees invited to participate, 25 consented. Four declined, four did not respond after several attempts of contact and one was on long-term sick leave. Our final sample included 16 ‘implementers’ – those directly tasked with developing and implementing the CAZ, 3 elected district councillors and 2 town councillors (unpaid elected members who serve a smaller, local parish), 2 business representatives (including taxi drivers) and 2 campaigners. Sixteen were female and fifteen were male. Of those recruited via snowball sampling, three had professed views in favour of the CAZ, and three had professed views in opposition to the CAZ.

**Fig. 1:**
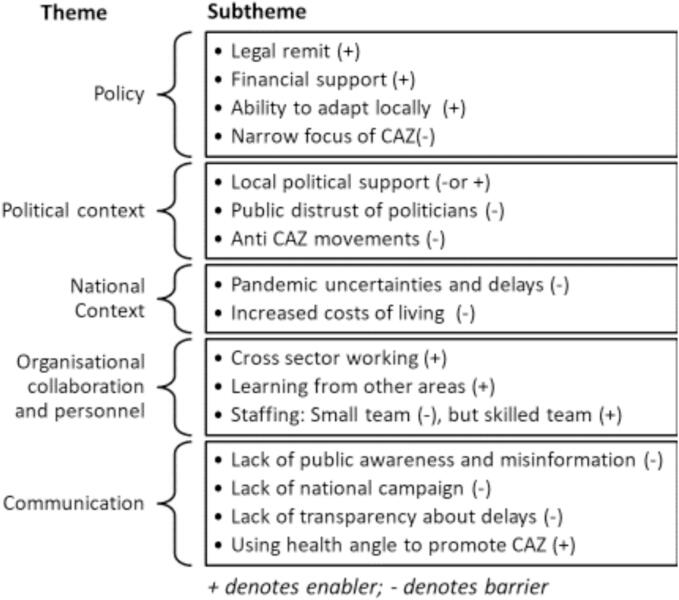
Key themes and sub-themes emerging from interviews.

Fig. 1 summarises the key barriers and enablers, grouped according to themes which were identified inductively from the analysis. These are described in further detail below. Where quotes are provided, we indicate whether they are from implementers, councillors (elected and non-elected), or campaigners.

### Key themes

#### Policy

##### Legal remit

Bradford Council were served a legal directive from the UK government to act, and this was arguably the most important enabler to policy implementation. The direction required the council to develop and model a range of CAZ options to bring pollution to compliant levels, and a Class C + charging CAZ (which included more stringent emissions standards for taxis registered in the city) was subsequently approved. This legal remit meant that there was no choice but to implement it, despite some opposition.

*“At the end of the day, we have been directed by the government to introduce the zone to improve air quality, so there’s no getting out of it, we need to do it,” (Implementer).*

##### Financial support

In addition to the legal diktat, Bradford Council was given over £40million to implement the CAZ, with approximately £31 million of this ear-marked for grants to support local small and medium businesses/companies to upgrade vehicles to help mitigate against potential economic impacts for those who may have non-compliant vehicles.

*“it’s the funding to support it, you can’t just do that [implement a CAZ] and then expect all the local people to take the hit, you’ve got to fund it,” (Implementer).*

*“We’ve got more mitigation funding for a CAZ of this type than anywhere else,” (Implementer)*

However, not all interviewees felt the level of funding was sufficient to allow stakeholders to upgrade their vehicles.

*“what we’ve noticed probably in the last two years is the value of second-hand vehicles has gone up significant, so you know, what we were offering in terms of grant funding, looking at it now it doesn’t seem very competitive,” (Implementer).*

##### Local adaptation

With the overall CAZ framework, there was some flexibility for adaptation, such as the charge levels, availability of grants and exemptions to meet local circumstances.

*“…as Government things go, [it’s] a really good process, they’ve allowed us to develop it locally so that it’s to our needs,” (Implementer).*

While it originally was thought Bradford would need a CAZ D, which includes private vehicles, during the early stages of development, those involved expressed concern about the potential impact having a detrimental impact on low-income families, along with the fear of public outcry. The team modelled many additional measures that could reduce emissions to avoid charging private cars, finally settling on a Class C CAZ plus raising minimum standards for registered private hire vehicles and taxis (referred to subsequently as Class C +).

Flexibility was also extended to setting the daily charges and grants procedures. After discussion with taxi drivers − a significant lobby group − the daily charge for non-compliant taxis was dropped from £12.50 to £7. The grants process was altered to provide drivers money in advance rather than retrospectively so they could afford to buy compliant vehicles.

*“what we’ve done is just tried to accommodate them and … [show], that we do listen and accommodate those people and make it so it’s not harder for them that it needs to be,” (Implementer).*

##### Limited scope of the CAZ framework

The parameters outlined in the CAZ Framework were felt by some to provided limited scope for activities and ambition. A narrow remit was allowed: targeting NO_2_ emissions only; a solo focus on transport and not on other sources or types of pollution (e.g., domestic heating and wood burning stoves).

*“I’m very aware that air quality isn’t just transport, so that’s my bug bear about all this, that it’s just focused on one source. So you’re only going to get so much of a reduction,” (Implementer).*

*“the focus is on business vehicles, not private cars, and yet … all our modelling shows they’re (diesel cars) 50 % of the problem,” (Implementer).*

Government funding was available to bring levels of NO_2_ down to the legal limit of 40 µg/m3 and no further. For example, in models, a CAZ C + would get NO_2_ down to legal levels, therefore other measures that would further reduce levels, such as electric buses, were not eligible for funding. Some expressed frustration that they were not able to be more ambitious in their plans to reduce pollution beyond the legal limits.

*“Basically they want you to do the least you can to get to the legal limit and they’ll pay for that, any more than that and they’re not interested,” (Implementer).*

*“Surely as a Council we’re not just going to say, well, that was dangerous, and now it’s a percent lower so it’s in orange now, not red, and therefore we don’t need to bother because that to me is not good enough,” (Implementer).*

#### Political context

##### Local political support

The political context was seen by many of the interviewees as a key challenge. There was concern the public would not like the CAZ, with consequences for local councillors.

*“You’re not going to embark on that without some calculation of the potential electoral upsides and downsides,” (Councillor).*

It was thought by many implementers that the public believed the CAZ was a choice that Bradford Council had decided to implement, not a legal requirement. One councillor mentioned:

*“This is all on [the Council]. The Government have just said that you need to improve your air quality. They haven’t said how to do it. It is the Councils’ decision to have a CAZ,” (Councillor).*

This meant extra efforts were needed to explain the legal obligation, need and benefits of a CAZ to the public, and all communications needed political approval:

*“People forget that we work in a political environment and that, particularly for marketing and communications, caused massive issues,” (Implementer).*

##### Public distrust of Government

General distrust of politicians was seen as another problem. One interviewee felt it did not matter to the public which party was bringing in the CAZ as levels of distrust of politicians were so high that no one would see the intervention in a positive light.

*“government generally has got such a bad reputation that anything government does has a bad reception, … there are all sorts of people who just feel, oh it’s an imposition and there’s some ulterior motives and it’s a way of increasing taxes,” (Campaigner).*

##### Anti-CAZ movements

During the interview period, there was national media coverage of anti-CAZ demonstrations in the neighbouring city of Manchester, and on the 6th of February, the local Mayor in Manchester announced that their CAZ would be indefinitely postponed (BBC, 2022). One respondent who was a local councillor said that Manchester’s campaign encouraged them to object to Bradford’s CAZ. Other cities, including Manchester and Leeds, were frequently cited by interviewees who opposed Bradford’s CAZ as evidence that it was, incorrectly, a choice rather than a Directive, for Bradford Council to implement the charging CAZ.

*“other cities have introduced Clean Air Zones without the need for a charge, obviously it’s probably too early to say what the impact of those has been on the air quality, but it can be done… and the Bradford Council have decided to make it a charging zone,” (Councillor).*

One implementer felt the negativity had become the dominant discourse surrounding the CAZ implementation, rather than the reduction of air pollution.

*“we’re at a stage now where I have to be honest, it’s not really about improving air quality in the shortest possible time, it’s about implementing a Clean Air Zone with the least reputational risk damage.”*

#### National context

##### Uncertainties and delays caused by the covid pandemic

The covid-19 pandemic impacted on many aspects of the CAZ implementation including operational aspects and acceptability. The justification for the Bradford CAZ was based on modelling of pollution levels before the pandemic, and what would be needed to get these to legal levels. According to one implementer, the pandemic resulted in a dramatic change in road transport, with a 50% drop at the start of UK lockdown restrictions (mid-March 2020), leading to reduced pollution levels. However, some implementers reported that was followed by higher traffic levels, and therefore pollution levels, as lock down restrictions eased.

The pandemic meant that assumptions considered during the development period, for example, the number of vehicles that would be upgraded in advance of the zone, and the composition of vehicles on the roads were felt to be less reliable. Additional modelling work had to be undertaken to look at long-term covid impacts on traffic.

The pandemic also impacted on the availability of staff to development systems needed for the CAZ (notably communications and information technology), key equipment to implement the CAZ (for example, the availability of automatic number plate recognition cameras), and availability of compliant vehicles. It was reported that some businesses which accessed a grant had difficulties in getting a new vehicle.

*“we’d seen from sort of all areas really, not just taxis, but LGV’s and HGV’s that the vehicle supply isn’t really there at the moment for them to upgrade that quickly,” (Implementer).*

In response, a ‘sunset’ period was implemented, whereby vehicles owners who had claimed a grant and ordered a vehicle would not have to pay a charge if the vehicle was not delivered before the CAZ went live.

##### Increased costs of living

Costs of living increased for many families during the pandemic. As well as increasing costs of fuel and other basic goods, the cost of compliant vehicles increased. However, the amount of grant subsidy available to help businesses upgrade did not increase from that originally planned.

*“we go into a vicious pandemic that knocks everyone sideways economically and suddenly what looked like an affordable change to a low emissions vehicle becomes a bit of a big ask,” (Implementer)*

The cost-of-living crisis, following on from the pandemic, was frequently stated by those in opposition to the CAZ as a reason it should be postponed or scrapped altogether.

*“[friends have said that] doing this is actually screwing us both ways because you’re taking money off businesses that work in the area and you’re making us pay more for our food,” (Implementer).*

#### Organisational collaboration and personnel

##### Cross-sector working

The multi-disciplinary, cross sector working board was felt to be a key enabling factor influencing both the development and implementation of the CAZ. It enabled staff across the council to have knowledge of the project, learn of capacity to deliver, gain knowledge of what was being done well, what needed improving and how best to build new capacity. Several interviewees felt the board had been very useful.

*“are there any lessons that you’ve learnt through this process that you think can help other sites when [they instigate] a Clean Air Zone? (interviewer)*

*Yeah, I mean it’s an obvious one in terms of having that wide cross cutting different services coming to that table around” (Implementer).*

##### Learning from other areas

Some of the implementers felt they had greatly benefited from speaking with other cities that were implementing CAZs, so avoiding potential pitfalls. Bradford implementers were in continuous talks with two other cities who had already launched their CAZs. They were keen to share their experiences with those from other cities so they too could benefit from the learning.

*“What we ended up with in terms of our plans and our preparation was a lot better because we used their experience to learn from, actually make sure Bradford didn’t fall into the same sort of problems that others had,”*

*(Implementer).*

##### Staffing

A difficulty seemed to have been a shortage of staff to deal with the development of the CAZ. People spoke of there being just three or four main members of staff within the early stages of CAZ development, with the result that there was a lot of *‘burning the midnight oil’*. An implementer suggested that, for future cities, the staff tasked with developing the zone from the start should have that as their sole job description, not as an addition to the day job.

The small team developing the CAZ received much praise from other interviewees in terms of commitment, knowledge, availability, and skills. It was felt they had ‘grafted’ and were very knowledgeable. They had engaged with dissenting councillors, campaigners, and stakeholders and ‘got them on board’. At the time of the interviews, over 80 % of taxis were compliant, compared to 5 % at the start of the process in February 2020 (Implementer). One implementer said they felt the process has taught them how to negotiate.

#### Communication

##### Lack of public awareness and misinformation

Getting information about the CAZ to communities was identified as problematic. At the time of the interviews, it was felt by several interviewees that few members of the public knew about the CAZ or what vehicles would be charged.

*“I think generally prior to the 1st of January [2022] it seems like there’s not been a lot of direct communication with the public, so they don’t seem to know about the Clean Air Zone,” (Implementer).*

The pandemic meant that the usual methods to help local businesses apply for grants, such as face-to-face workshops, could not happen, making it harder to reach those needing a grant. Furthermore, traditional methods of communication with the public (billboards, local radio, local papers) were no longer viable for informing residents of the approaching CAZ. There was no dedicated communications worker for the CAZ until January 2022 when the covid response was winding down. The perceived lack of awareness of the CAZ in the pre-implementation period allowed misinformation about the CAZ to spread.

*“I think the biggest hurdle that we've come across is lack of information out there or a lot of misinformation out there, where people have made up their own assumptions and then it’s rife on social media,” (Implementer).*

Misinformation ranged from referring to the charge as a ‘tax to line the council’s coffers’ to the future charging of private cars.

##### Lack of a national campaign

Part of the Councils’ remit was to advertise the CAZ up to an hours’ drive away, taking in other urban areas such as Sheffield and Manchester who were also planning to implement CAZs. Some implementers stated that there had originally been plans for a national campaign, managed centrally, to raise awareness of all the CAZs being planned across the UK, and the reasons for their implementation. However, the national campaign did not happen, raising concerns amongst implementers that many businesses coming into Bradford from outside the area would get fined due to lack of awareness, which would increase anti-CAZ sentiment.

*“some businesses could go to Bradford and Manchester and Sheffield all in one day, or in a week they could easily go into three Clean Air Zones for example, so I think the general awareness of Clean Air Zones and why they’re having them, and what businesses can do could be, you know, increased,” (Implementer).*

Without a national campaign, there were difficulties in coordinating advertising between the CAZ cities. There were also added complications to work out the wording that would be considerate to Manchester, where CAZ plans were postponed, or Leeds, which was no longer operating a CAZ, while not diminishing the CAZ in Bradford (Implementer).

***“****How do we publicise the Bradford Clean Air Zone in a sympathetic way to Manchester? It’s just horrendous,” (Implementer).*

##### Lack of transparency about delays

The launch date of the CAZ was substantially delayed, due in part to difficulties caused by the pandemic. Dates for launching across the country were set in partnership with the Government and chosen based on the readiness of the city implementing the CAZ, and to stagger the timings of different city launches. Bradford was originally due to launch the CAZ in October 2021, then January 2022, then ‘Spring 2022′. The actual launch date of the 26th September 2022 was not confirmed until the 21st July that year. At the time of the interviews (January – April 2022), there was therefore a high level of uncertainty about when it would happen.

Road information signs had been erected in autumn 2021, stating the launch date was January 2022. The signs had to be covered or altered to read ‘Spring 2022′ when the date was postponed. Not being able to specify a revised date was felt to be damaging to public confidence in the scheme and was felt to make the council look incompetent.

*“It just looks like you’re not being transparent if you’re not stating dates and it’s just literally because we don’t know it, which also doesn’t look very good neither, but and you know, that’s all to do with the government process,” (Implementer).*

##### Using health angle to promote the CAZ

From the start, publicity about the Bradford CAZ stated that a key reason for implementation was to improve the health of the population. Most implementers felt that framing the CAZ in terms of the positive health impacts was a key factor in facilitating its acceptance. One implementer felt that further demonstrating the link between health and poverty is what ‘*sold*’ the CAZ to reluctant councillors (Implementer). In addition, *“no-one can argue about children’s health… it feels to me as though that argument has really helped us,”* (Implementer). It has allowed the CAZ to be presented in a positive rather than negative light. Even those in opposition to the CAZ made it clear they were not against reducing air pollution to improve health; rather they disliked the method being used.

In publicity, the health information was directly derived from Bradford’s own health statistics and from a local research programme (McEachan et al., 2024) which was felt to have an impact:

*“So people sometimes look at health data and they sort of like go yeah, but that’s somewhere else, whereas we’ve got health data that’s says no, it’s our children in Bradford,” (Implementer).*

A small number of interviewees were sceptical that the health message had had such a strong impact. It was felt by them that the publics’ automatic reaction was to consider the economic impact rather than the public good.

*“there’s only a few that say well, it’ll be good because the air pollution at Saltaire roundabout were shocking. There’s only a few that are linking the benefit of cleaner air to the zone,” (Implementer).*

### Interpretation of findings: What were key barriers and enablers?

Table 1 describes the barriers and enablers observed through the complementary lens of Pfadenhauers contextual (2017) and McTigue’s implementation (McTigue et al., 2020) theories. Of these, three key contextual (legal, political and epidemiological) and three key implementation (policy remodelling, conflict/opposition/ambiguity, and collaboration) dimensions are highlighted below.

**Table 1:** Interpretation of barriers and enablers during the implementation phase of the Bradford CAZ. (+) denotes an enabler; and (−) denotes barrier.

**Dimension**	**Bradford example**
**Pfaundenhauer’s (2017) Context dimensions**	
Legal	(+) UK Government is under a court ruling to reduce NO_2_ to legal levels. Central Government created a legal requirement for certain local authorities to implement the policy. This gave implementers the power to enforce the policy regardless of dissent.
Political	(+) Implementing the CAZ was a political directive from central government to local government which enabled its realisation. (−) Political opposition from different parties and individuals at different stages hindered progress (for example, the motion to suspend the CAZ on the 15th March 2022, the anti-CAZ campaigning in local elections), and affected media discourse about the policy, seen in local and national press. (−) High profile change of policy in Manchester to delay implementation of their CAZ, and the less well known cancelling of the CAZ in Bradford’s neighbour Leeds, possibly led to reduced confidence in local CAZ plans amongst key stakeholders. (−) Political sensitivities meant it was difficult for the implementation team to communicate about the CAZ.
Epidemiological	(+) There are high levels of respiratory illness and vulnerable populations (e.g. young and old, and those living in deprivation) in Bradford. A local longitudinal birth cohort study provided a pipeline of relevant evidence to support message that pollution impacted children’s health. The health issue was used as a justification for implementing the CAZ and included in key messaging.
Socio-economic	(−) Levels of deprivation in Bradford are high, and there was concern about the potential impact of the CAZ on low-income groups. In response, a CAZ C was implemented to avoid charges to private vehicles, reducing impact on low-income car owners. (−) There is a strong likelihood that covid and the cost of living crisis impacted on affordability and availability of compliant vehicles. (−) The national financial crisis possibly led to increased fears of economic impact of CAZ, or increased focus of economic impact rather than health impact.
Socio-cultural	(−) There was generally a lack of trust in authorities. (−) Covid pandemic altered the perceived long term mode and pattern of travel behaviours within the city, including fluctuations in car users, public transport users, and the number of delivery vehicles, leading to questions about whether the CAZ was still required.
Ethical	(+) The aspiration is for CAZ is to improve the health of Bradford residents. The policy follows the ‘polluter pays’ principle. (+) By implementing a CAZ C category, rather than CAZ D, the aim was to minimise potential negative impact on low income communities. (+) Grants were available for local businesses to help upgrade vehicles and exemptions are available for local residents. (−) Implementers were frustrated that they were not able to access funding to do more to reduce pollution as the focus was on achieving only legal limits.
Geographical	The shape of the CAZ was dependent on air pollution statistics relating to roads & topography; generally, the areas of the city with high pollution levels were more deprived areas.
**McTigue’s (2020) implementation theory**	
Policy remodelling	(+) Working within a tight framework, some local adaptability was allowed in certain policy areas, resulting in Bradford’s CAZ C category (which avoided taxing private vehicles), its shape, charges and grants available. (+) After consultation, concessions to taxis to reduce the charge from £12.50 to £7 may have helped to prevent large scale protest.
Opposition, conflict and ambiguities	(−) There was opposition to CAZ from different local political parties at different times in the process. Actors within both main political parties tried to avoid ownership of the policy at times. (−) A number of opposition party councillors campaigned on an anti-CAZ platform. The local elections were used as an anti-CAZ platform was used by a handful of potential councillors in local May 2022 elections. (−) Lack of clear national campaign, perceived lack of awareness, or misinformation meant that general public were not clear what the zone entailed.
Collaboration and interaction between those involved in the policy process	(+/-) Highly structured interaction between Bradford local authority, and Government in development of plans and launch date of CAZ, with both pluses and minuses in terms of occasional ‘jumping through hoops’ creating more work, and creating a detailed plan that considers many aspects and provides local flexibility. (+) Informal beneficial interactions between various local authorities developing CAZ plans. (+) Bradford local authority worked with businesses affected, especially taxi/private hire sector and bus companies, to ensure policy on the ground would work, possibly resulting in lower levels of opposition. (+) The core development team ‘grafted’ to get things done to a time line, they were skilful in listening, negotiating and compromising, keeping taxis and buses on their side.
Policy document	(−) The CAZ Framework originally published in 2017 set out guidance for local authorities to use to create a clean air plan to reduce air pollution in the quickest possible time. The framework referred to ‘charging’ and ‘non-charging’ clean air zones as options, however there was ambiguity about what non-charging clean air zones involved, how they might work, or whether they were valid options. In Bradford this was used by some to oppose the plan, saying that a charging CAZ was optional. In October 2022 the CAZ framework was updated and the term ‘non-charging’ CAZ was removed. (−) The CAZ framework focused on reduction of traffic-related NO_2_ emissions only. This meant other activities to reduce pollution from other sources could not be included. (+) The creation of a detailed plan by Bradford Council, which was approved by Government was a prerequisite to obtaining funds to implement the CAZ.
Availability of resources	(+) Funds of ∼£40 million were made available from central government to implement the Bradford Clean Air Plan and CAZ. This included approximately £31 million grants for local residents and businesses to upgrade vehicles. (−) The amount of grant funding available to local residents and businesses to upgrade vehicles was perceived as modest and only able to cover a small proportion of the costs involved upgrading vehicles. Most taxis upgraded to hybrid models and not electric. (−) There was a lack of staff in early stages of development.
Policy champions	(+) Small, dedicated core development team committed to getting the CAZ through, taking it from start to launch. (−) There were policy ‘non-champions’ in opposition who were very vocal. (−) No high profile public figure emerged backing the policy.
Intra-organisational support and communications	(+) The developed of the Bradford Clean Air Plan was led by an Air Quality Board with members from diverse departments including public health, transport, planning and research. This enabled board to maximise opportunities for added health impact. (−) Complex IT requirements led to delays. (−) All communications had to be signed off by political leaders, leading to delays in release of communications.
Characteristics of an organisation	(−) Procedures around approving communications, procurement and grant giving rules, public consultation, and pre-election purdah regulations meant some activities could not be completed quickly. (−) Staff shortage within communications and IT teams led to delays. (−) The CAZ development team did not have the authority to require all departments to work with them when they requested.
Bureaucratic power	(−) A national campaign was felt to be necessary to raise awareness of the location and types of CAZs. It is beyond the means of individual local authorities to coordinate a national advertising campaign to raise awareness of the location of CAZs and their categories, but the campaign has not been forthcoming from central Government.

#### Contextual influences

***Legal:*** Having a legal remit from the Government was a key enabler of policy implementation: the local authority was legally required to implement a CAZ and there was therefore no choice but to proceed. This gave them a strong defence against opponents.

***Political:*** Local political dissent, particularly within the context of local elections was a key barrier. Dissent mostly focussed on the fear of economic stagnation in an already deprived city. The opposition party within the local authority (part of the same political party as the Government who introduced the CAZ framework) were some of the most vocal opponents of the CAZ implementation in Bradford, using it as a local election campaign and also submitting a (ultimately unsuccessful) motion to suspend the Bradford CAZ on the 15th March 2022. Much resource and time was spent dealing with the political motions and working to counteract vocal negative opinion.

**Epidemiological:** At the time of interview, all implementers were aware of the harmful impacts of pollution. A local longitudinal cohort study provided evidence of the harmful impacts of pollution on children’s health. The implementers defended the CAZ by communicating the health benefits of reducing air pollution and using the city’s own health statistics. They perceived this to be a key enabler as it allowed a strong justification for the CAZ.

#### Implementation factors

**Policy remodelling:** Whilst there was a stringent framework to work within there was scope for local adaptation and this was seen as a key enabler There was real concern about the impact of charging private vehicles on low-income families. Implementers were able to show that a class C CAZ, combined with minimum hybrid standard for all registered taxis or private hire vehicles, would be likely to reduce pollution to compliant levels. Other adaptions included reducing the daily charge for taxis and local exemptions. These adaptions were felt to increase acceptability.

**Opposition, conflict and ambiguities:** Several implementers felt that a negative perception of the CAZ was the dominant discourse as the political conflict was amplified in local media, resulting in the CAZ being framed as ‘divisive’ to the public. Added to this were other media platforms used by those vehemently opposed to the CAZ and where misinformation thrived and could not be controlled by the Council. There was no national communication campaign, as a result respondents felt there was not sufficient widespread knowledge of the CAZ and this had allowed a space for negative misinformation to spread. These conflicts and ambiguities damaged the confidence of local politicians tasked with leading implementation.

**Collaborations and interactions:** Implementation was led by a collaborative multi-disciplinary team. Cross-department working allowing alignment of health and environment agendas within the plans to maximise co-benefits. Transparent and collaborative communications with other local authority areas meant the team were able to learn from pitfalls experienced by other local authority areas. Close interactions with key stakeholders, including local businesses, helped to shape the policy and mitigations which was felt to increase the acceptability of the CAZ and soothe opposition.

## Discussion

This research explored barriers and enablers to the implementation of a Low Emission Zone in a large UK city at a key moment in its development phase. Key barriers included the political context, conflict and opposition. Key enablers included collaborative working, ability to adapt the policy and availably of local evidence to justify the policy. To our knowledge this is the first study of its type exploring in rich detail the barriers experienced in the real time implementation of a LEZ within a large city. We found striking similarities in the barriers identified to those found in other major transport and urban policies such as initiatives to promote health and climate co-benefits (Negev et al., 2022) and public transport (McTigue et al., 2020). Thus, the learning reported here is likely highly relevant to other transport related policy in addition to LEZs. We offer recommendations to others areas implementing this type of transport policy in Box 1.

Wider literature identifies political will and leadership to be a crucial factor in implementing urban policies to improve health and climate outcomes (McTigueet al., 2020, Negevet al., 2022). Here, we found political influences simultaneously operating as an enabler, providing a legal basis for implementation, and a barrier, as destabilising local political conflicts took hold. Understanding how to navigate these types of socio-political contexts will be crucial for the successful realisation of LEZ and wider transport policy. However these contexts are often neglected in urban and transport research (Kębłowskiet al., 2022, Negevet al., 2022). We therefore encourage transport researchers to embrace a ‘critical’ perspective that recognises the complexity of diverse social, political and economic drivers which impact on policy success (Kębłowski et al., 2022).

Like others, we found the LEZ to be a highly divisive policy (Christiansen, 2018). The vocal anti LEZ campaign groups and political campaigning against the LEZ charges damaged the confidence of the implementers tasked with enacting the policy, and led to beliefs that the policy was not supported by members of the general public. However, these ‘loud’ voices may not be representative of public opinion. A previous study conducted with Bradford residents (N = 1949) just prior to the interviews found that 70 % were supportive of a LEZ in principle (Mebrahtu et al., 2023). Communicating the benefits of LEZ more effectively can help to bolster public acceptability, leading to increased political support and confidence. Messages which are positively framed, relevant for local contexts, and clear on the health and climate co-benefits of policy are likely to have most impact (Riley et al., 2021). In the current context, implementers highlighted the importance of having local evidence about the relationships between pollution and health, which they used to justify the policy. Strong ‘policy champions’ (McTigue et al., 2020) will be crucial to promoting acceptability. However, given the increasing scepticism and mistrust of politicians highlighted here, and elsewhere (Hudson et al., 2019) we would recommend consideration be given to identify credible and trusted actors within communities such as health professionals, research institutions, and citizen associations (Riley et al., 2021).

Collaborative, cross-sector working was a key enabler. Previous authors have highlighted entrenched ‘siloed’ working which means that the potential health and climate co-benefits of transport related policy are not maximised (Barneset al., 2018, Hudsonet al., 2019, Negevet al., 2022). Here, having members from diverse disciplines including public health, transport and planning enabled the planning board to maximise opportunities for added value. For example, incorporating research (e.g. Rashid et al., 2021) and engagement (e.g. Islam et al., 2022) in the development of the plans enabled implementers to justify requests for the maximum available funding for grants to allow taxi drivers and businesses to upgrade vehicles.

Implementers recognised the ultimate aim of the LEZ as to improve health, and this was seen as a critical organising and motivating force. However, there was frustration that focus was on legal compliance and that measures were approved (and funded) only to get the city down to legal levels, and not lower. Others have noted that “air pollution policies are often aimed solely at bring areas in compliance with standards, and neglect the fact that health is of the upmost importance” p5, (Nieuwenhuijsen et al., 2023) To maximise the health co-benefits of transport policy we need to rethink the issue with a systems perspective − one which crosses disciplinary boundaries, and brings together diverse stakeholders from planning, health and climate with politicians and communities. Bringing these groups together will be key to driving transformational change within cities. But this is not without its challenges. Research which aims to shed light on the complexity of the systems in which these types of policies operate, including competing interests and short-term political decision making cycles (Nieuwenhuijsen et al., 2023) is needed to aid decision making.

### Strengths and limitations

Our study had several strengths. Respondents included those at the ‘coalface’ of implementing the policy, as well as local politicians, and campaign groups. This gave us rich insights into the process of implementing the policy. The interviews were at a point where there was substantial uncertainty, conflict, and opposition to the policy. Real time research provides an opportunity to explore some of these factors, identifying critical problems and solutions that come apparent in the sticky process of implementation (Institute for Government, 2011). We followed best practice guidelines in conducting and reporting our research (Tong et al., 2007). Our study is one of the first to explore in detail barriers to implementing LEZ with key decision-makers and implementers, and adds to the small, but growing evidence base in this area. Given the increase in popularity of LEZ across Europe and continuing expansion of the LEZ framework in the UK our findings are highly relevant to other areas.

There were some limitations. Many of the stakeholders we interviewed were involved in implementing the LEZ; this may have introduced bias in that these respondents may have had more positive views. To ensure a more balanced view, our purposive sampling technique also ensured inclusion of key groups who opposed the LEZ. Participants were assured of anonymity at the start of each interview, and the wide views expressed (both positive and negatives) suggest that participants were comfortable giving their opinions. Future research could usefully focus on more representative methods, for example surveys to gain opinions for a wider group of stakeholders. We focused on one city in the process of implementing a LEZ, and different barriers and enablers may be found in other areas.

Box 1 **: Key lessons learned.**

**For those implementing a LEZ locally:**

• Create a multi-disciplinary team to lead the planning and implementation process. Ensure that leads from key functions including health, planning, transport, legal, human resources, and IT are included. Ensure that research, monitoring, and evaluation is included as a core component of this team.
• Engage with key stakeholder groups, including communities and local business, from the start. Listen to their needs and adapt policies where it is viable to do so. Pay attention to perceived potential unintended or unanticipated consequences, including for low-income groups. Explore mitigations to these potential adverse impacts, for example, by providing grants to facilitate upgrading of non-compliant vehicles and exemptions.
• Ensure there is adequate staffing resource at early stages of policy development, to fully consult and engage with communities and businesses.
• Engage with other areas implementing LEZ to share learning around successes and challenges.
• When communicating about the LEZ, focus on the health and climate benefits of reducing pollution. If possible, use local evidence on pollution and health that are directly relevant to the communities in your area.
• Recognise that dissent, opposition, and misinformation will polarise opinion. Provide clear, and repeated information to the public and other stakeholders about the zone, including who will be charged, and what help is available.
• Identify a local and trusted policy champion(s) who can communicate the reasons for the intervention. Use a range of media to spread messages.
• Work with local research partners to develop plans to monitor and evaluate the impact of the LEZ on pollution, transport related behaviour and health.

**For central policy makers:**

• LEZ policy should aim to improve health, and not focus on legal compliance levels. There is no safe level of pollution.
• The ability to adapt LEZ frameworks to local areas is a strength.
• However, having different types of LEZ in different areas is confusing for the public. A national awareness campaign to explain the need for the LEZ and where they are located would help to increase knowledge of potential health and environmental gains, and reduce the number of people being unwittingly fined.
• Ensure that the robust systems level evaluation is in place to understand the health and environmental benefits of the LEZ, along with unintended consequences.

### Conclusion

Low emission zones, whilst increasing in popularity across Europe, remain contentious. In the UK we describe the challenges experienced by one urban area tasked with developing and implementing a low emission zone, in real time, six months before it was launched. The political context, conflict and opposition were key barriers. Highly vocal opposition groups whose voices ‘shouted the loudest’, other areas delaying their LEZ and a general distrust of Government and politicians reduced the confidence of implementers. Key enabling factors included collaborative working, combining expertise from multiple disciplines including health and research; and the ability to adapt policy to suit local contexts. Using the health angle to justify implementation was seen as an important strategy. Implementing transport policy is complex, future research needs to take a systems perspective which recognises the complexity of socio-political and temporal contexts in which policy is embedded. Ultimately the proof will be in the pudding: do these policies reduce pollution and improve health? To understand this is it will be important to ensure that policies are implemented as intended, and in place for long enough to track immediate and longer-term health and economic impacts.

## Funding

This report is independent research funded by the National Institute for Health Research (NIHR) Public Health Research, NIHR 128833 − Evaluating the life-course health impact of a city-wide system approach to improve air quality in Bradford, UK: A quasi-experimental study with implementation and process evaluation. RM is supported by the NIHR Yorkshire and Humber Applied Research Collaboration (NIHR200166). The views expressed in this publication are those of the author(s) and not necessarily those of the National Institute for Health and Care Research or the Department of Health and Social Care.

RM and MB are supported by the UK Prevention Research Partnership (MR/S037527/1), an initiative funded by UK Research and Innovation Councils, the Department of Health and Social Care (England) and the UK devolved administrations, and leading health research charities. Weblink: https://mrc.ukri.org/research/initiatives/prevention-research/ukprp/.

## CRediT authorship contribution statement

**Cathy Knamiller:** Writing – review & editing, Writing – original draft, Project administration, Methodology, Investigation, Formal analysis. **Rukhsana Rashid:** Writing – review & editing, Project administration, Methodology, Investigation, Formal analysis, Conceptualization. **Maria Bryant:** Writing – review & editing, Supervision, Methodology, Funding acquisition, Conceptualization. **Emma Bailey:** Writing – review & editing, Formal analysis. **Rosemary R.C. McEachan:** Conceptualization, Funding acquisition, Methodology, Supervision, Writing – original draft, Writing – review & editing.

## Declaration of competing interest

The authors declare that they have no known competing financial interests or personal relationships that could have appeared to influence the work reported in this paper.

## Data Availability

Data will be made available on request.

## Footnotes

1 The least restrictive class, CAZ A, charges non-compliant taxis and buses; a class CAZ B also includes heavy goods vehicles; a class CAZ C extends the charging further to include light goods vehicles. The most restrictive, CAZ D charges all non-compliant vehicles, including private vehicles.
