# Supplementary material for: Who shouts the loudest? A qualitative study exploring barriers and enablers to implementing a low emission zone in a Northern UK city
Source: Transp Res Interdiscip Perspect. 2024 May;25:None. doi: 10.1016/j.trip.2024.101112 (PMC11184590; doi:10.1016/j.trip.2024.101112)
Supplement: Supplementary Data 1 [file mmc1.docx]

**Supplemental File**

**Interview discussion guide (v2 05.01.22). Approved by Bradford Leeds NHS research ethics committee (Approval ref: 20/YH/0158)**

***Note: B-CAP:*** *Bradford Clean Air Plan; CAZ: Clean Air Zone*

B-CAP development

1. What support / advice did you receive from the Government when developing the B-CAP
   1. Was there anything else that you would have liked support with?
   2. Was there anything that you found helpful or unhelpful?
2. How well do you think you are prepared for the BCAP including the CAZ?

*Prompt*

- 1. What measures have you have to put in place?
  2. Did you have enough time/ information to prepare?
  3. Was support available?
  4. What support did you need?

1. Were there any options that were originally considered as part of the plans that have not been introduced that you think should have been? (provide context ie Scoot, cycle lanes, improved infrastructure).

Marketing and public consultation

1. Were you involved in how the BCAP was being introduced to the public?
2. How and when was the public consulted?
3. Do you think the plans are acceptable to the public?

1. What more can be done to improve acceptability?
2. What worked well/ didn’t work well with public consultation?
3. How did the BCAP team respond to public opinions during the development of the plan?

Implementation

1. What has worked well and what didn’t work well with the rollout?

Covid 19 pandemic

1. How did the pandemic affect your plans for the BCAP?

*Prompt*

1. Unexpected delays
2. Customers accessing grants etc.
3. Date for rollout
4. Communication with the public
5. To what degree is the BCAP as you expected?

*Prompt*

- 1. What impact do you think the CAZ will have?

1. How long will the CAZ in the BCAP remain in place?

*Prompt*

- 1. Should there be a time limit?

1. To what degree is the BCAP as you expected?

*Prompt*

- 1. Didn’t affect you much
  2. Had more effect that expected
  3. What about the CAZ?

1. Do you think the BCAP goes far enough? Or too far?
2. What else could have been included in the BCAP?
   1. Is there anything you would have preferred not to have been included?
3. What do you think about the pricing plan? (provide details)
   1. Did this influence some of the plans you put in place?
   2. How was this formulated? Were you a part of the pricing plan?
   3. What do you think about the potential penalties for non-compliant vehicles? Are they enforceable? What was the response from the public after consultations?

Recommendations

1. What else would you have liked to see included in the BCAP?
   1. Is there anything you would have preferred not to have been included?
2. Do you think the BCAP has been a good investment?
3. Why?
4. How could it have been improved?
5. What would you do differently?
6. Is there a longer term sustainability plan?

Health impact

1. Do you think the measures will help improve air quality in Bradford?
2. Have you noticed a change already?

If no, do you think a change will happen? When?

*Prompt*

1. Have you noticed a change in peoples transport/ travel behaviours
2. Has this been positive?
3. Do you think the BCAP will have a positive impact on people’s health overall?
4. Is there anything else you would like to share with us about the CAZ or other aspect of the clean air plan?
